# A national cohort study (2000–2018) of long-term air pollution exposure and incident dementia in older adults in the United States

**DOI:** 10.1038/s41467-021-27049-2

**Published:** 2021-11-19

**Authors:** Liuhua Shi, Kyle Steenland, Haomin Li, Pengfei Liu, Yuhan Zhang, Robert H. Lyles, Weeberb J. Requia, Sindana D. Ilango, Howard H. Chang, Thomas Wingo, Rodney J. Weber, Joel Schwartz

**Affiliations:** 1 Gangarosa Department of Environmental Health, Rollins School of Public Health, Emory University, Atlanta, GA USA; 2 Department of Epidemiology, Rollins School of Public Health, Emory University, Atlanta, GA USA; 3 School of Earth and Atmospheric Sciences, Georgia Institute of Technology, Atlanta, GA USA; 4 Department of Biostatistics and Bioinformatics, Rollins School of Public Health, Emory University, Atlanta, GA USA; 5 School of Public Policy and Government, Fundação Getúlio Vargas, Brasília, DF Brazil; 6 Department of Epidemiology, School of Public Health, University of Washington, Seattle, WA USA; 7 Department of Neurology and Human Genetics, School of Medicine, Emory University, Atlanta, GA USA; 8 Department of Environmental Health, Harvard T.H. Chan School of Public Health, Boston, MA USA

**Keywords:** Alzheimer's disease, Environmental impact

## Abstract

Air pollution may increase risk of Alzheimer’s disease and related dementias (ADRD) in the U.S., but the extent of this relationship is unclear. Here, we constructed two national U.S. population-based cohorts of those aged ≥65 from the Medicare Chronic Conditions Warehouse (2000–2018), combined with high-resolution air pollution datasets, to investigate the association of long-term exposure to ambient fine particulate matter (PM_2.5_), nitrogen dioxide (NO_2_), and ozone (O_3_) with dementia and AD incidence, respectively. We identified ~2.0 million incident dementia cases (*N* = 12,233,371; dementia cohort) and ~0.8 million incident AD cases (*N* = 12,456,447; AD cohort). Per interquartile range (IQR) increase in the 5-year average PM_2.5_ (3.2 µg/m^3^), NO_2_ (11.6 ppb), and warm-season O_3_ (5.3 ppb) over the past 5 years prior to diagnosis, the hazard ratios (HRs) were 1.060 (95% confidence interval [CI]: 1.054, 1.066), 1.019 (95% CI: 1.012, 1.026), and 0.990 (95% CI: 0.987, 0.993) for incident dementias, and 1.078 (95% CI: 1.070, 1.086), 1.031 (95% CI: 1.023, 1.039), and 0.982 (95%CI: 0.977, 0.986) for incident AD, respectively, for the three pollutants. For both outcomes, concentration-response relationships for PM_2.5_ and NO_2_ were approximately linear. Our study suggests that exposures to PM_2.5_ and NO_2_ are associated with incidence of dementia and AD.

## Abstract (web summary)

Air pollution has been linked to neurodegenerative disease. Here the authors carried out a population-based cohort study to investigate the association between long-term exposure to PM_2.5_, NO_2_, and warm-season O_3_ on dementia and Alzheimer’s disease incidence in the United States.

## Introduction

Dementia is a major public health issue, affecting >47 million people worldwide^1^. Alzheimer’s disease (AD) contributes to about two-thirds of dementia cases and is the sixth leading cause of death in the United States^2^. In response, the National Alzheimer’s Project Act was signed into law to overcome dementia, and the National Plan was launched with Goal 1 aiming to prevent and effectively treat dementia (delay onset, slow progression) by 2025^3^. As there are no disease-modifying treatments for the most common types of dementia, it is a top research priority to identify modifiable risk factors for dementia that can be intervened on at the population level.

There is growing evidence associating air pollution with neurodegenerative disease. A systematic review by Peters et al.^4^ found nine longitudinal studies of air pollution and AD and related dementias (ADRD). Among them, five of six showed a positive association between increased exposure to PM_2.5_ and dementia or AD; four of four showed an association between NO_2_ and dementia or AD, whereas one of three did so for ozone (O_3_). Fu and Yung^5^ published a review and meta-analysis of AD and air pollution, and found a twofold excess risk of AD for a 10 µg/m^3^ increase of PM_2.5_ among six studies, and no increased risk for NO_2_ in four studies, nor for O_3_ in three studies. There have been several longitudinal studies since these reviews, with the majority finding positive associations between air pollutants and either dementia or AD^6–14^. A few of these studies examine the associations in US populations, and these studies have almost exclusively used hospitalization as a measure of morbidity^6,7,11,13^. The diagnosis of ADRD, however, likely occurs in doctor visits, and ADRD does not generally result in hospitalizations. Thus, hospitalization records may not well represent either disease incidence or prevalence in the overall population, and likely leads to an underestimation of the number of cases, and unclear generalizability. In addition, neuropathologic changes are known to occur many years prior to the diagnosis^15^, and the relevant time window in which air pollution might increase the risk of dementia or AD is unclear.

To address these knowledge gaps in studying ADRD incidence in the US, here we constructed a national, population-based cohort study from Medicare data to investigate the impact of long-term exposure to PM_2.5_, NO_2_, and warm-season (May to October) O_3_ on dementia and AD incidence. To better measure disease incidence, we required a 5-year “clean” period without events of interest after enrollment in Medicare system and used all Medicare claims nationwide (2000–2018), including inpatient and outpatient claims, carrier file (primarily doctor visits), skilled nursing facility, and home health-care claims to identify the first diagnosis of ADRD. We used high-resolution (1 km × 1 km) daily surface-level concentration fields of PM_2.5_, NO_2_, and O_3_ for 2000–2016, estimated based on ground observations, satellite data, chemical transport modeling, land use, and meteorological data using national spatiotemporal ensemble exposure models^16–18^. We assigned air pollution exposure to subjects based on resident ZIP code, and calculated time-varying 5-year lagged moving averages for each follow-up year.

## Results

### Study population characteristics

Table 1 provides descriptive information on the dementia cohort and AD cohort. Both cohorts were followed after requiring a 5-year period without events of interest to better capture disease incidence. There were 12.2 and 12.4 million people in dementia and AD cohorts, respectively (Table 1). Most of the studied subjects (78.5% and 78.1% for dementia and AD, respectively) entered the cohorts between ages 65 and 74. The median follow-up was 7 years in both cohorts. More than 90% were not eligible for Medicaid, indicating that most were defined as being above the poverty level^19^. A majority of the study population had comorbidity at some point during follow-up. 16.6% developed dementia (~2.0 million cases), 6.5% developed AD (~0.8 million cases), and Supplementary Table 1 presents detailed demographic information for the cases and non-cases.

**Table 1:** Descriptive statistics for the study population.

	Dementia cohort		AD cohort	
Variables	Number	%	Number	%
Number of events	2,025,130	16.6	804,668	6.5
Number of the total population	12,233,371	100	12,456,447	100
Total person-years	89,035,081	100	93,278,266	100
Median follow-up years	7	7		
*Age at entry (years)*				
65–74	9,597,788	78.5	9,734,481	78.1
75–114	2,635,583	21.5	2,721,966	21.9
*Sex*				
Male	5,023,879	41.1	5,107,942	41.0
Female	7,209,492	58.9	7,348,505	59.0
*Race*				
White	11,023,202	90.1	11,214,287	90.0
Black	649,081	5.3	666,619	5.4
Other^a^	561,088	4.6	575,541	4.6
*Medicaid eligibility*				
Dual-eligible	800,139	6.5	852,499	6.8
Non-dual eligible	11,433,232	93.5	11,603,948	93.2
*Comorbidity*				
Diabetes	4,433,314	36.2	4,590,000	36.8
Hypertension	10,273,506	84.0	10,502,180	84.3
Stroke	1,991,730	16.3	2,137,239	17.2
Heart failure	3,388,540	27.7	3,598,028	28.9
No comorbidities^b^	1,642,674	13.4	1,865,751	15.0
*Air pollutants*^c^				
Annual PM_2.5_ (µg/m^3^)	9.3 (3.2)	9.3 (3.2)		
Annual NO_2_ (ppb)	17.1 (11.6)	17.1 (11.6)		
Warm-season O_3_ (ppb)	42.6 (5.3)	42.6 (5.3)		

^a^Other included Asian, Hispanic, American Indian, or Alaskan Native, and unknown.

^b^Means none of the above comorbidities.

^c^Presented as mean concentration (interquartile range).

### Air pollution levels

**Fig. 1:**
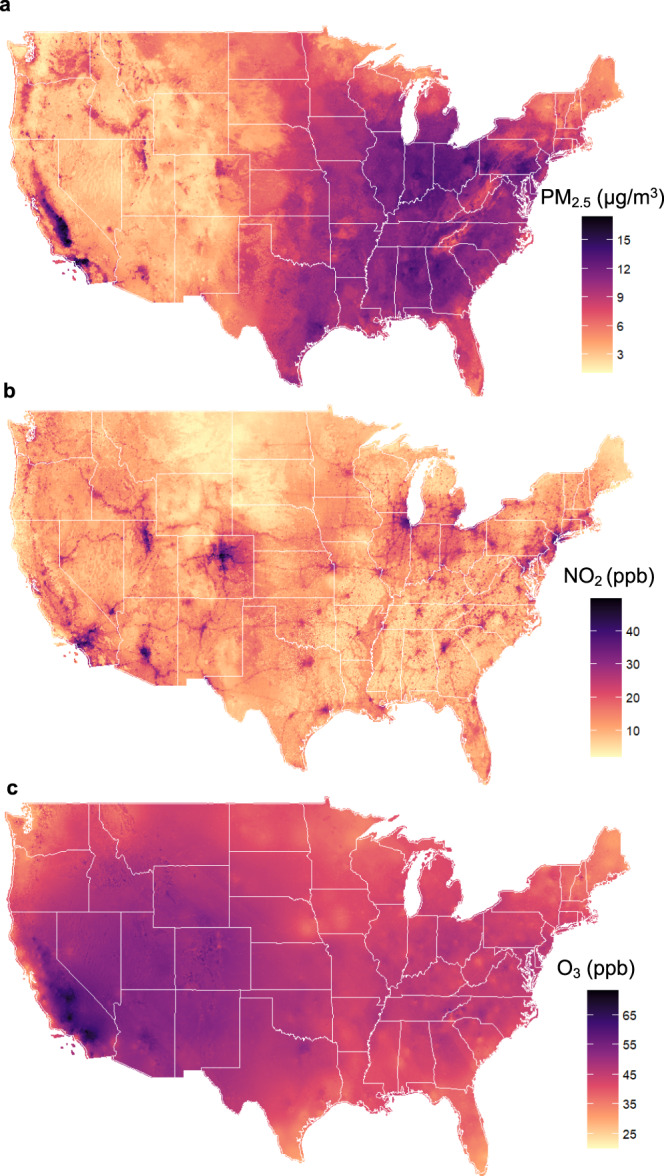
Maps of the spatial distributions of air pollutants studied. The three panels present the average concentrations of **a** annual PM_2.5_ (μg/m³)^16^, **b** annual NO_2_ (ppb)^17^, and **c** warm-season O_3_ (ppb)^18^ at 1-km^2^ resolution across the contiguous United States over the study period, respectively. Map was made from the census bureau shapefile (cb_2017_us_county_500k.shp, https://www2.census.gov/geo/tiger/GENZ2017/shp/) using R software, and no licenses are required as this map was provided free of any copyright restrictions. Source data are provided as a Source Data file.

The average annual level of PM_2.5_ of cohort participants during the study period, 9.3 µg/m^3^, was below the US EPA standard of 12 µg/m^3^; The average NO_2_ level was 17.1 ppb, considerably below the EPA annual standard of NO_2_ of 53 ppb. The annual warm-season average O_3_ was 42.6 ppb. EPA does not have a standard for annual warm-season O_3_. As a reference, the EPA standard for daily maximum of 8-hour average O_3_ is 70 ppb (Table 1). We examined warm-season O_3_, because O_3_ is more readily formed in the warm season^20^, and this metric is often used in long-term epidemiological studies^21^. Fig. 1 shows the distribution of the three pollutants across the US during our study period, as estimated by the exposure models used in our analysis. PM_2.5_ is highest in the eastern US and in California, O_3_ in the West, and NO_2_ (largely produced by traffic) in urban centers. Further detail on exposure levels can be found in Supplementary Table 2. The three pollutants in our data were only modestly correlated. The Pearson correlations between pollutants (average exposure within the past 5 years) were as follows: PM_2.5_ and O_3_ 0.22, NO_2_ and O_3_ 0.19, and NO_2_ and PM_2.5_ 0.39.

### Health effect estimates

**Fig. 2:**
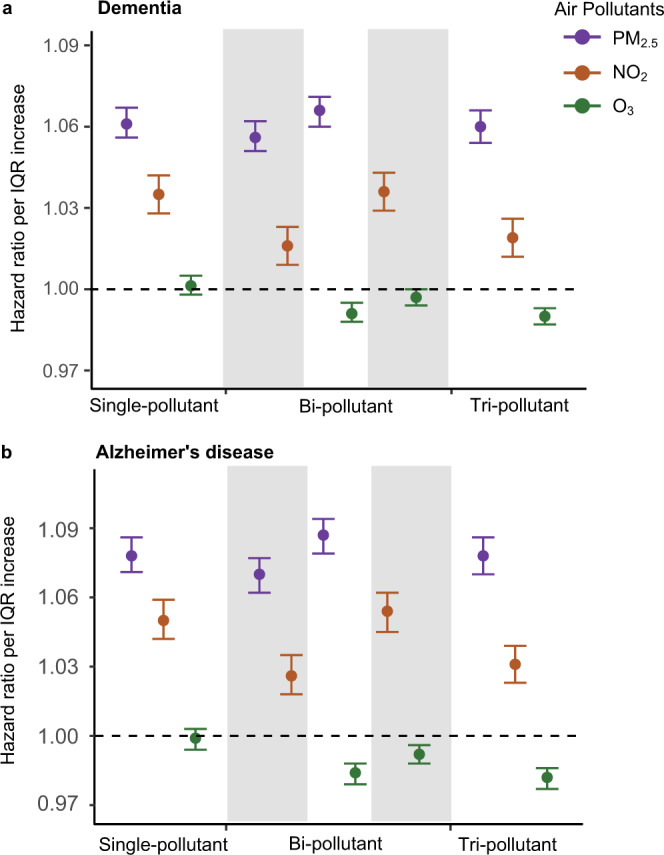
Results of the Cox proportional hazards models. The two panels present the hazard ratios of **a** dementia (*n* = 12,233,371 individuals examined) or **b** Alzheimer’s disease (AD, *n* = 12,456,447 individuals examined) associated with per IQR increase in annual PM_2.5_, or annual NO_2_, or warm-season O_3_ concentration, respectively. The estimated hazard ratios were obtained using single pollutant, bi-pollutant, and tri-pollutant models. Error bars stand for the 95% confidence intervals. The gray and white stripes are used to distinguish any two adjacent models. Source data are provided as a Source Data file.

Fig. 2 provides the main study results from the Cox proportional hazards models stratified by individual characteristics, adjusting for neighborhood-level socioeconomic status (SES) (see details in Methods), behavioral risk factors, health-care capacity variables, and residual temporal and spatial trends (see Methods). An interquartile range (IQR) increase in the 5-year average of the annual PM_2.5_ (3.2 µg/m^3^) in the 5 years prior to diagnosis was associated with an increased risk of dementia (HR = 1.061, 95% CI: 1.056, 1.067) in single-pollutant models, which changes little in models with other pollutants. An IQR increase in 5-year average NO_2_ (11.6 ppb) is associated with an HR of 1.035 (95% CI: 1.028, 1.042) in single-pollutant models, dropping to 1.019 (95% CI: 1.012, 1.026) in multi-pollutant models. An IQR increase in the 5-year average of warm-season O_3_ (5.3 ppb) has little effect on dementia rates, with HRs of 1.002 (95% CI: 0.998, 1.005) in single-pollutant models and 0.990 (95% CI: 0.987, 0.993) in multi-pollutant models.

The findings for AD have a similar pattern to those for dementia, but the hazard ratios (HRs) are higher per IQR increase, being 1.078 (95% CI: 1.071, 1.086) for PM_2.5_, 1.050 (95% CI: 1.042, 1.059) for NO_2_, and 0.999 (95% CI: 0.995, 1.003) for O_3_ assessing each pollutant individually. After adjusting for co-pollutants, the effect estimates were similar for PM_2.5_ (HR = 1.078, 95% CI: 1.070, 1.086) and attenuated for NO_2_ (HR = 1.031, 95% CI: 1.023, 1.039), while O_3_ is slightly protective (HR = 0.982, 95% CI: 0.977, 0.987).

### Concentration–response relationships

**Fig. 3:**
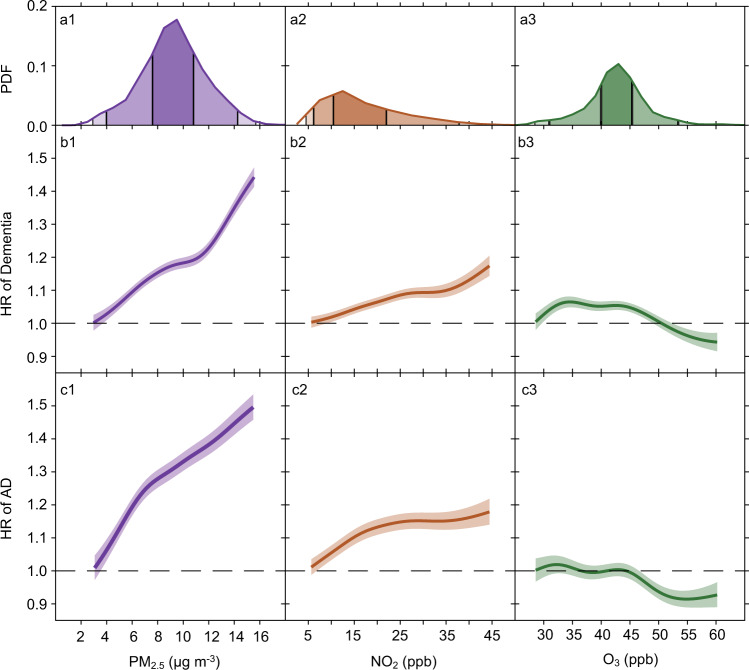
Concentration-response curves. Panel. **a** presents the probability distribution functions (PDF) of long-term PM_2.5_, NO_2_, and O_3_ exposures; Panel **b** presents the concentration–response relationships between each pollutant and dementia; Panel **c** presents the concentration-response relationships between each pollutant and Alzheimer’s disease (AD). The concentration–response curves, derived from the tri-pollutant models, are shown for the concentration ranges between 0.5th to 99.5th percentiles of the pollutants, i.e. with 1% poorly constrained extreme values excluded. Shading areas (from the darkest to the lightest) in **a** represent pollutant concentration ranges of the IQR (i.e., 25th to 75th percentiles), 95% (2.5th to 97.5th), and 99% (0.5th to 99.5th), respectively. Source data are provided as a Source Data file.

Figure 3 presents penalized spline curves for the three pollutants, derived from the tri-pollutant models. The concentration–response (C-R) relationships for PM_2.5_ are approximately linear for both dementia and AD across the exposure distribution, although for AD there is a suggestion of a steeper slope below 8 µg/m^3^. For NO_2_, the C-R curves for dementia and AD are linear for low concentrations (<25 ppb), and then level off for higher concentrations. The curves for O_3_ are essentially flat for both endpoints until high, and rarely occurring concentrations. These results suggest that the adverse effects of PM_2.5_ and NO_2_ on dementia or AD are at least retained, if not strengthened, at low levels of air pollution exposure (e.g., below the WHO air quality guidelines: PM_2.5_ ≤ 10 μg/m^3^, NO_2_ ≤ 20 ppb). Across the 0.5th to 99.5th percentile of the exposure distribution, PM_2.5_ shows the strongest effect on dementia or AD among all pollutants.

### Effect modifications

**Fig. 4:**
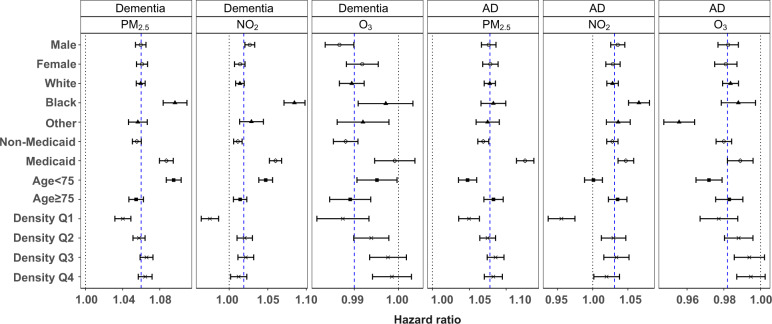
Effect modifications by sex, race, Medicaid eligibility, age, and population density. Results represent the hazard ratios of dementia (*n* = 12,233,371 individuals examined) or Alzheimer’s disease (AD, *n* = 12,456,447 individuals examined), from the tri-pollutant models, per IQR increase in 5-year average PM_2.5_, NO_2_, or O_3_. Error bars stand for the 95% confidence intervals. The blue dashed lines indicate the overall effect estimates for all groups. “Other” includes Asian, Hispanic, American Indian or Alaskan Native, and unknown. “Density Q1–Q4” denotes quartiles of population density, i.e., low population density, low-medium population density, medium-high population density, and high population density. Source data are provided as a Source Data file.

We examined five potential effect modifiers (sex, race (white, Black, other), Medicaid eligibility, urbanicity (expressed in quartiles of population density), and age (<75, ≥75). Fig. 4 shows HRs in each subgroup, based on the interaction term between exposure and the potential effect modifier. Most marked results were seen for an increased hazard of dementia and AD for Black individuals compared with white individuals in relation to both PM_2.5_ and NO_2_; a similar pattern was found for those eligible for Medicaid. At the same time, those living in the rural areas (i.e., lowest quartile of population density) were found to have notably lower effect estimates between both dementia and AD and both PM_2.5_ and NO_2_. Regarding age, those <75 had a markedly stronger association between dementia and both PM_2.5_ and NO_2_, while the association was stronger between AD and both PM_2.5_ and NO_2_ among those older than 75. Finally, we found little evidence of an interaction between PM_2.5_ or NO_2_, and sex in relation to dementia or AD. For O_3_, all subgroup-specific estimated HRs were below one, and the association with both endpoints was stronger among those not eligible for Medicaid or those living in rural areas. The *p* values for testing the null hypothesis that the estimated associations are the same between subgroups classified by a subpopulation indicator are shown in Supplementary Table 3.

### Sensitivity analysis

Associations between long-term exposure to PM_2.5_, NO_2_, O_3_, and dementia or AD were robust to a series of sensitivity analyses. First, a more strict “clean period” by excluding anyone who had a diagnosis for dementia or AD in their first 10 years of follow-up yielded results similar to the main analyses (Supplementary Table 4). Second, based on this new subcohort (with 10-year clean period), the use of alternative exposure windows (annual exposure 10, 5, 1, or 0 years prior to disease diagnosis, i.e., lags 10, 5, 1, or 0) all support a positive association with PM_2.5_ and NO_2_, but not O_3_, though HRs varied in magnitude (Supplementary Table 4). For both outcomes, associations with PM_2.5_ and NO_2_ were attenuated with increasing lag periods. Third, the observed associations with dementia or AD were not mediated by nor modified by comorbidities, such as diabetes, hypertension, stroke, and heart failure (Supplementary Table 5). Fourth, to account for potential bias related to moving of the resident address, we performed analyses for subjects who did not move during the follow-up period (i.e., non-mover cohort). The results are roughly consistent with the main analysis, showing significant positive associations for PM_2.5_ and NO_2_, but not for O_3_, for both dementia and AD. However, compared with the main model, the effects of PM_2.5_ are attenuated and the effects of NO_2_ are enhanced (Supplementary Table 6). At last, we assessed the effect of possible outcome misclassification in two ways, one via using a linear regression model based on rates, and the other based on prior estimates of Medicare sensitivity and specificity and estimating the true number of cases within strata. Both methods support the findings from our main analysis, i.e., long-term exposure to PM_2.5_ and NO_2_, but not O_3_, were significantly associated with an increased incidence of dementia and AD; both also suggest that misclassification has biased our findings to the null (Supplementary Tables 7 and 8).

## Discussion

We found elevated HRs for both dementia and AD in relation to PM_2.5_, and less markedly to NO_2_, while HRs for warm-season O_3_ were not elevated. We did this study in a large US cohort (12 million), with national coverage, and including non-urban areas. For both PM_2.5_ and NO_2_, we found a larger effect on AD compared to dementia, which may reflect the fact that dementia includes a wide range of diseases with distinct etiologies, some of which may be unrelated to air pollution, while AD is a subset of dementia and a single disease, for which we found a stronger association. On the other hand, we know of no pathophysiologic data, which indicate that air pollution might affect AD more than dementia; data relevant to disease mechanisms would suggest both AD and the broader category of dementia might be affected by air pollution^22,23^.

We also found that shorter time windows between exposure (PM_2.5_ or NO_2_) and disease showed slightly higher effect estimates, and we suggest (assuming the association is causal, although we acknowledge this is not possible to conclude from an observational study such as this) that this implies an acceleration of an existing process (dementia progression, i.e., accelerating cognitive decline that was already well developed). Further research assessing specific aspects of progression is warranted in the future, preferably with data on the stage of dementia at diagnosis. Moreover, our diagnosis free period requirement provides reasonable assurance that we are looking at incidence, and use of physician’s visits, nursing home data, etc. to ascertain diagnosis avoids missing large numbers of cases, possibly not missing at random, which likely occurs in studies using diagnoses based on hospital admission records.

Some of our models showed a protective effect of O_3_. However, when we compare results in Fig. 2, we see that in single-pollutant models the effect estimate for O_3_ was null, whereas in bi-pollutant models with either PM_2.5_ or NO_2_, the effect size for O_3_ was pushed below the null (albeit not significantly) and only in the tri-pollutant model was it protective at the conventional 0.05 level. Moreover, in the bi-pollutant models with O_3_, the effect sizes for PM_2.5_ and NO_2_ increased from their level in the single-pollutant models. We interpret this as evidence that there is no effect of O_3_, and the protective effect seen in the tri-pollutant model may be due to collinearity, even though the correlations between pollutants are moderate. The Pearson correlation between 5-year moving average NO_2_ and warm-season O_3_ is positive, which is inconsistent with previous studies within cities that show a negative correlation^24^. One possibility is that our study included exposure estimates with full coverage including both urban and rural areas, unlike previous studies that only focused on cities. The chemical regime of O_3_ formation in rural areas is typically NO_x_-limited, and a positive correlation between O_3_ and NO_2_ is expected.

Higher effect estimates were observed for more populated areas (Fig. 4). PM_2.5_ in urban areas consists of more ultrafine particles, such as soot and metals, mainly emitted from traffic sources. Numerous studies have shown that traffic-related air pollution can be a risk factor for dementia and AD^25–27^. Ultrafine particles can move up the olfactory nerve into the brain directly and can penetrate into the blood and reach the blood–brain barrier^28^. Although NO_2_ is a single species and the composition is uniform everywhere, its spatial pattern shows a high correlation with major highways and cities, and the NO_2_ concentration may serve as a proxy for other traffic/urban air pollutants, which might have high neurotoxicity.

Our results are broadly consistent with developing literature, which shows relatively consistent effects for PM_2.5_ and NO_2_, but less consistent for O_3_. We observed an HR of 1.06 for dementia and an HR of 1.08 for AD per 3.2 μg/m^3^ increase in annual PM_2.5_ in single-pollutant models, i.e., equivalent to an HR of 1.10 and an HR of 1.13 per 5 μg/m^3^ increase in PM_2.5_. These values can be compared with our previous Medicare cohort study using hospitalizations^7^, reporting an HR of 1.06 for dementia and an HR of 1.17 for AD per 5 μg/m^3^ increase in annual PM_2.5_. A cohort study conducted in Ontario, Canada by Chen et al.^29^ simultaneously accessed the effects of PM_2.5_, NO_2_, and O_3_ on dementia risks, and they also found significant associations with PM_2.5_ and NO_2_, but not O_3_. Recent 2018 and 2020 Lancet Commission overviews of modifiable environmental agents associated with disease noted a possible association between air pollutants and dementia, but noted the evidence still preliminary^1,30^.

The epidemiologic findings are supported by brain imaging and toxicologic studies. Regarding brain imaging, Shaffer et al.^31^ have found associations between PM_2.5_ and AD neuropathology upon autopsy, while Laccarino et al.^32^ found an association between PM_2.5_ and positive positron emission tomography (PET) scans for amyloid. Younan et al.^33^ followed 1000 women and found increased cognitive decline on immediate memory/new learning, and increased MRI-determined risk for future AD using a neuroanatomical risk score. These recent findings support earlier neuroanatomical associations found by others^34,35^. Toxicological studies support several plausible biological mechanisms. PM_2.5_ has been consistently linked to oxidative stress, neuroinflammation, systemic inflammation, and all of which, in turn, have been reported as key pathways to AD pathogenesis^26,34,36^. Magnetite nanoparticles from combustion processes have been discovered in the human brain, indicating that particles from urban air pollution can reach the blood–brain barrier (e.g., by interacting with dysfunctional cells)^37^.

The strongest relationship we found with both endpoints was for PM_2.5_ among the three pollutants. If the US PM_2.5_ levels could be lowered by 3.2 μg/m^3^, which is the IQR, then the attributable fraction (AF) of dementia and AD due to current exposure levels, based on our main results from tri-pollutant models assuming a linear relationship, would be 6% and 7%, respectively. Namely, if there is a causal relationship (although we acknowledge this is not possible in the present associative study), this would suggest an estimated 6% of dementia cases and 7% of AD cases would potentially be avoided if PM_2.5_ levels decreased by 3.2 μg/m^3^, which is approximately the difference between our large cities like New York and Chicago and smaller cities like Portland, Buffalo, or Baltimore^38^. Likewise, if the US NO_2_ levels could be reduced by 11.6 ppb (IQR), an estimated 2% of dementia cases and 3% of AD cases would be avoided assuming a causal relationship.

Our study has several strengths. To our knowledge, this is the first nationwide, population-based cohort study that focuses on the simultaneous health effects of PM_2.5_, NO_2_, and O_3_ on dementia and AD. The large sample size gives us ample power to detect effects even though they are small, which is often the case in environmental studies. Second, the use of Medicare claims data that include doctor’s visits rather than restricting the data to hospitalizations is likely to include many more cases, given that many cases are never hospitalized, and also cases which are diagnosed earlier and hence better reflect incidence. Evidence can be found by comparing recent data in another paper about dementia and AD hospitalization in Medicare data^7^, to the data in the current paper. To allow for a fair comparison, we used the same inclusion/exclusion criteria and restricted to the same time period (2000–2016) and geographic region (i.e., the lower 48 states), and we found that using just hospitalization missed nearly 90% of dementia cases and 60% of AD cases, compared to using our current data including doctor’s visits (Supplementary Table 9). Third, we used a conservative method by requiring a 5-year “clean” period and restricting the analysis to subjects with continuous enrollment in Medicare Fee-for-Service (FFS), and Part A (hospital insurance) and Part B (medical insurance) programs throughout the study period, which can ensure that cases were newly diagnosed and thus better approximate incidence. At last, we were able to control for a large number of individual- and neighborhood-level covariates. The inclusion of comorbidities had a negligible effect on our results, suggesting that they are unlikely mediators in our studied associations. However, a formal mediation analysis would be important to confirm these findings.

Despite these advantages, some key limitations should be noted. One limitation, typical of using administrative records to identify disease, is potential misclassification of the outcome. AD cases in our database represented only ~40% of the dementia cases, suggesting important under-ascertainment of AD, given that AD represents ~60–80% of dementia cases^2^. This percentage is quite similar to the findings of Goodman et al.^39^, who found that AD represented 44% of all dementia diagnoses in Medicare data in 2013, including both hospitalizations and doctor visits. It is likely that a large number of our dementia cases, who show no AD diagnosis in Medicare, actually had AD, but physicians did not feel confident to make the more specific diagnosis. This is supported by the findings of Taylor et al. (2009), who compared Medicare data to clinical diagnoses considered as the gold standard, and found that the sensitivity of dementia was 0.85 but was considerably lower, 0.65, for AD. At last, given the long insidious onset of dementia and lack of information on the stage of dementia at diagnosis, our study may mismeasure the year of onset of dementia, though we applied the 5-year “clean” period as inclusion criteria to reduce the bias.

We have assumed that outcome misclassification is non-differential (conditionally independent of exposure to air pollutants, conditional on confounders); there are no data indicating otherwise. We have conducted two types of sensitivity analyses to adjust for such misclassification of classifying dementia or AD cases as without dementia or AD (false negative, or 1-sensitivity), and the misclassification of non-dementia, non-AD subjects to one of the diseases (false positive, or 1-specificity). Both these methods of adjustment for false negatives and false positives were in agreement that our results were likely to under-estimate the true HRs for PM_2.5_ and NO_2_ for both dementia and AD.

Another limitation of our study is the potential exposure error and its spatial pattern, although the exposure prediction model we used has excellent predictive accuracy^16–18^. Using larger scale ambient air pollution assigned to individuals has been shown to have a net bias towards the null, consistent with non-differential measurement error, which reflects some degree classical type of error^40–42^. In addition, our study is subject to unmeasured and residual confounding. While we were able to control for a number of potential confounders at the neighborhood level, we had no individual-level data on SES and education, a limitation implying some mismeasurement of confounders, which may have biased our results (moderately, given that these unmeasured confounders are not likely to act as very strong risk factors for dementia), in an unknown direction. Furthermore, we only studied the Medicare FFS population who enrolled in both Part A and Part B programs, precluding generalizability to the entire US elderly population. Further work is also needed to determine if the association is generalizable in other countries.

Our study provides evidence that long-term exposure to PM_2.5_ mass is associated with increased ADRD incidence. Future studies of air pollution and dementia in other countries, including low-to-middle-income countries on which there are currently few studies, will be important. Understanding the potential bias and unmeasured confounding, given the limitations of observational studies, is encouraged. Examining the role of specific pollutant components in ADRD may also be important because different components of PM_2.5_ (e.g., metals, elemental carbon, organic carbon, sulfate, and nitrate) and different sources of PM_2.5_ (e.g., traffic, industrial, cooking, and biomass burning) may have different neurotoxicities. A better understanding of component-specific and source-specific effects of PM_2.5_ on ADRD could potentially inform pollution control policies on specific sources.

## Methods

### Study population

Data were drawn from the Medicare denominator file and the Medicare Chronic Conditions Warehouse (CCW), both from the Centers for Medicare and Medicaid Services (CMS). In the U.S., people are eligible to enter the Medicare program after they turn 65 years of age. The denominator file (i.e., the enrollment file) contains enrollment records for all Medicare beneficiaries, including age, sex, race, Medicaid eligibility (a proxy for SES), the date of death (if any), and ZIP code of residence. Age, Medicaid eligibility, and ZIP code of residence are updated annually. CCW provides the date of the first occurrence with dementia or AD diagnosis code across the available Medicare claims. Based on these two Medicare databases, we constructed an open cohort including all Medicare beneficiaries aged 65 and over who were always enrolled (1) in Medicare Fee-for-Service program; and (2) in both Medicare Part A (hospital insurance) and Part B (medical insurance) in the contiguous United States between 2000 and 2018. These criteria excluded those with any time in Medicare Advantage (HMO) over the study period since claim records are not available for these beneficiaries and excluded those only enrolled in Medicare Part A or Part B. If we relaxed these restrictions to broaden the cohort, the chance of missing dementia or AD cases among those additional people brought into the analysis would be high.

We created separate data sets for dementia and AD. For each cohort, we further required a “clean” period of 5 years after enrollment, during which there were no diagnosis codes for dementia or AD. For example, a participant entering Medicare in 2005 would be required to be dementia-free until 2010; follow-up for disease incidence began only then. By removing potentially prevalent cases in their first five years of follow-up, a diagnosis after that “clean” period more likely approximates disease “incidence”. We considered that 5 years was a reasonable period to ensure that a person truly was not demented prior to the Medicare diagnosis; however, we also explored a 10-year clean period in sensitivity analyses. Therefore, study subjects entered the cohort on January 1st of the year following the “clean” period and were followed until the first diagnosis of the outcome of interest across all Medicare claims, death, or end of follow-up. We excluded this 5-year clean period from follow-up time to avoid immortal time bias. A schematic diagram of the cohort selection criteria is illustrated in Supplementary Fig. 1. Our research is approved by Emory’s IRB (#STUDY00000316) and the Centers for Medicare & Medicaid Services (CMS) under the data use agreement (#RSCH-2020-55733). The Medicare dataset was stored and analyzed in Emory Rollins School secure cluster environment (HPC), with Health Insurance Portability and Accountability Act (HIPAA) compliance.

### Data management and maintenance of confidentiality

Emory provides and operates the secure cluster environment HPC certified for use with confidential health records data (e.g., Medicare) storage and analysis and safeguard the data in compliance with the HIPAA security rule. The CMS data files are stored on the secure servers at Emory Rollins School of Public Health. All the participating research members are granted Emory-affiliated user accounts with which to access processed data for the duration of the project. No transfer of CMS data from Emory systems is allowed for any user. In addition, a technical data scientist at Emory is monitoring the activities of the user accounts and manually inspecting any derived data and output from analyses. When an analysis is complete, data access will be removed for that investigator.

### Outcome classification

The primary outcomes of interest for this study were time to (1) all-cause dementia and (2) AD subtype. CCW includes pre-defined indicators for dementia and AD, which are identified using an algorithm^43^ that incorporates information from all available Medicare claims (such as inpatient and outpatient claims, Carrier file, skilled nursing facility, and home health-care claims) indicating that an individual was diagnosed with dementia or AD (ICD-codes provided in Supplemental Material^43^). This algorithm applied by Medicare to define dementia and AD is primarily based on Taylor et al.^44^ and Taylor et al.^45^. CCW provides the date of the first occurrence with the dementia or AD diagnosis code. In the dementia cohort, the outcome dementia was defined as the first occurrence of a diagnosis code of dementia, while for the AD cohort, AD was defined as either (1) the first occurrence of a diagnosis code of AD with no prior diagnosis of dementia, or (2) the first occurrence of a diagnosis code of dementia when there was a subsequent diagnosis code of AD (under the supposition that the original dementia diagnosis was probably AD, given the subsequent AD diagnosis).

### Exposure assessment

High-resolution daily ambient PM_2.5_ (24-hour average), NO_2_ (1-hour maximum), and O_3_ (8-hour maximum) concentrations at 1-km spatial resolution for the entire United States were derived using spatiotemporal ensemble models that integrated three different machine learning algorithms, including neural networks, random forest, and gradient boosting. The ensemble-based model was calibrated using hundreds of predictors, including satellite measurements, chemical transport model simulations, land-use terms, meteorological variables, and monitoring measurements from the Environmental Protection Agency (EPA) Air Quality Systems (AQS). This ensemble learning approach yielded strong prediction model performance for each pollutant, with an average cross-validated coefficient of determination (*R*^2^) of 0.89, 0.84, and 0.86 for annual mean PM_2.5_, annual mean maximum 1-hour NO_2_, and warm-season (May to October) mean maximum 8-hour O_3_, respectively^16–18^. We averaged these 1-km resolution predictions for each pollutant at the ZIP code scale across each year^46^, because ZIP Code is the smallest level of geography in the Medicare data. We used the annual averages in each ZIP code, for each calendar year, as the exposure estimates for each Medicare beneficiary according to the ZIP code of residence. We calculated time-varying 5-year moving averages of exposure for each follow-up year for each subject. For example, air pollution estimates for someone entering Medicare in 2005 and being diagnosed with dementia in 2013 would include the average air pollution over the period from 2008–2013 and linked to the last follow-up year (i.e., 2013). All dementia and AD events were linked to exposures averaged over 5 years prior to diagnosis, and any annual residential mobility changes by ZIP code were taken into account, based on their yearly residence in the Medicare database. All high-resolution PM_2.5_, NO_2_, and O_3_ data used in this study were available from 2000 to 2016. For the follow-up year of 2017, we assigned the multiple-year average exposure during 2012–2016 to each subject (since 2017 exposure is not available); for the follow-up year of 2018, we assigned the multiple-year average exposure during 2013–2016 to each subject.

### Covariates

Individual-level age at entry, sex, race, and Medicaid eligibility were obtained from the Medicare denominator file. We also obtained neighborhood-level covariates in our study. These included ZIP code-level SES variables (population density, % Black population, education, median household income, % owner-occupied housing units, and % population above 65 years of age living below the poverty line), county-level behavioral risk factors (smoking rate and body mass index) and health-care capacity variables (number of hospitals and active medical doctors), as well as a geographical region. Specifically, SES variables were obtained from the 2000 U.S. Census^47^, 2010 U.S. Census^48^, and the American Community Survey for 2005–2012;^49^ behavioral risk factors were obtained from the Behavioral Risk Factor Surveillance System (BRFSS) between 2000 and 2016;^50^ and healthcare capacity data were obtained from 2010, 2015, and 2018 American Hospital Association Annual Survey Database^51^. We linearly interpolated or extrapolated any missing data based on the data available^52^. Data were also available for comorbidities (diabetes, heart failure, stroke, hypertension) in CCW. These covariates have been associated previously with ADRD and may be associated with air pollution, and hence were candidate confounders to be included in models^53,54^.

### Statistical analysis

We fit a series of stratified Cox proportional hazards models with a generalized estimating equation (GEE)^55^ to estimate the associations between long-term exposure to PM_2.5,_ NO_2_, and O_3_ on dementia or AD among the elderly, where the coefficient for the exposure variable was the parameter of interest, and years of follow-up was the time scale. Specifically, we fit single-pollutant, bi-pollutant, and tri-pollutant models and estimated HRs per interquartile-range (IQR) increase in the 5-year average of the annual PM_2.5_, NO_2_, and warm-season O_3_ concentrations in the 5 years prior to diagnosis. All three pollutants are of interest because some prior literature has shown associations between each of them and dementia^7,25,29,56^. GEE was used to adjust for residual autocorrelation within ZIP code with the use of robust standard errors (and 95% CIs). To allow for flexible strata-specific baseline hazard functions, we stratified all models on four individual characteristics, including sex, race (white, Black, other), Medicaid eligibility, and 1-year categories of age at study entry. To adjust for potential confounding, we included neighborhood-level SES, behavioral risk factors, and health-care capacity variables in our analyses. Potential residual temporal and spatial trends were controlled by respectively including a linear term for calendar years and indicator variables for the geographical region^7^.

To assess the shape of the C–R relationship between each air pollutant and dementia or AD, we respectively fit penalized splines^7^ for PM_2.5_, NO_2_, and O_3_, adjusting for all covariates included in the tri-pollutant models. To identify subpopulations who might be more vulnerable than others, we assessed potential effect modification by sex, race, Medicaid eligibility, age groups (aged 75+ vs. below 75), and urbanicity (quartiles of population density) on the multiplicative scale by including interaction terms between these potential modifiers and pollutants.

In addition, we estimated the attributable fraction (AF) of dementia and AD cases due to PM_2.5_ and NO_2_ air pollution, for those in the US exposed to an additional IQR of PM_2.5_ (a difference of 3.2 μg/m^3^) and NO_2_ (a difference of 11.6 ppb), beyond current levels in US cities with relatively low exposure (i.e., 7 µg/m^3^ for PM_2.5_ and 4 ppb for NO_2_, the counterfactual)^38^, using results from the multi-pollutant model, and using standard AF calculations when the entire population is exposed (RR-1)/RR (see Steenland and Armstrong^57^).

We conducted a series of sensitivity analyses to test the robustness of our main findings. First, we repeated the analyses using a “clean” period of 10 years, i.e., thinking that excluding cases with a diagnosis during their first 10 years of enrollment would increase the probability that we are capturing the first diagnosis and thus more closely estimating disease incidence, albeit at the cost of a smaller number of years of follow-up and cases. Second, using this new subcohort, we assessed alternative exposure time windows by comparing the results using different lags (0-, 1-, 5-, and 10-year lags), in which exposure was assigned either as the annual exposure at 10 years prior to case (or the risk set for given cases), or 5 years prior, or 1 or 0 years prior. We posit that if a shorter lag between exposure and disease fits the data best, assuming the association is causal, would imply an acceleration of an existing process (e.g., dementia progression) by air pollution, whereas a longer lag might indicate the air pollution has an effect in more initial stages of neurodegeneration (e.g., involved in the onset of dementia). In addition, to evaluate whether the associations we observe can be attributed to comorbidities also linked to air pollution, we additionally adjusted for the comorbidities (including diabetes, hypertension, stroke, and heart failure), and also restricted analyses to subjects without the comorbidities. Finally, we conducted analyses to estimate the effect of possible outcome misclassification in two ways. First, we fit linear regression models for the rate of dementia or AD (events/person-time) with a GEE, which in theory should target an approximately unbiased estimate of the additive effect^58^. Second, we considered the possible effect of outcome misclassification following methods similar to those described by Fox et al.^59^. We obtained estimates of misclassification parameters from Taylor et al.^45^ and adjusted the observed outcomes for each stratum to match up with the expected true values given pre-specified values for sensitivity and specificity for the outcome classification (details provided in Supplemental Material).

All computational analyses^60^ were run on the Rollins HPC Cluster at Emory University. R software, version 4.0.2, was used for all analyses. A two-sided *P* < 0.05 was considered statistically significant.

### Reporting summary

Further information on research design is available in the Nature Research Reporting Summary linked to this article.

## Supplementary information

Supplementary Information

Reporting summary

## Data Availability

Ensemble-based PM_2.5_ data that support the findings of this study are available from 10.7927/0rvr-4538, NO_2_ and O_3_ data are available from 10.6084/m9.figshare.16834390. Behavioral risk factors are available from https://www.cdc.gov/brfss/annual_data/annual_data.html; SES data are available from https://www.census.gov/data/datasets/2000/dec/summary-file-3.html, https://www.census.gov/data/datasets/2010/dec/summary-file-1.html, and https://www.census.gov/data/developers/data-sets/acs-1year.html; health-care capacity data are available from https://data.hrsa.gov/topics/health-workforce/ahrf. The rules governing the Medicare dataset prohibit any sharing of the health datasets being used for our epidemiologic research. Restricted by our Data Use Agreement with the US Centers for Medicare & Medicaid Services, the Medicare data that support the findings of this study are neither sharable nor publicly available. Academic and non-profit researchers who are interested in using Medicare data should contact the US Centers for Medicare & Medicaid Services directly to obtain their own datasets upon completion of a Data Use Agreement. Source data are provided with this paper.

## Source data

Source Data

## Code availability

The R codes for the epidemiological analyses are publicly available from 10.6084/m9.figshare.16843528.

## References

[1] Livingston G (2017). Dementia prevention, intervention, and care. Lancet.

[2] Heron, M. P. Deaths: leading causes for 2017. *Natl. Vital. Stat. Rep.* **68**, 1–77 (2019). 32501203

[3] Khachaturian ZS, Khachaturian AS, Thies W (2012). The draft “National Plan” to address Alzheimer’s disease-National Alzheimer’s Project Act (NAPA). Alzheimer’s Dement..

[4] Peters R (2019). Air pollution and dementia: a systematic review. J. Alzheimer’s Dis..

[5] Fu, P. & Yung, K. K. L. Air pollution and Alzheimer’s disease: a systematic review and meta-analysis. *J. Alzheimer’s Dis.* **77**, 701–714 (2020). 10.3233/JAD-200483 32741830

[6] van Wijngaarden E (2021). Neurodegenerative hospital admissions and long-term exposure to ambient fine particle air pollution. Ann. Epidemiol..

[7] Shi L (2020). Long-term effects of PM2· 5 on neurological disorders in the American Medicare population: a longitudinal cohort study. Lancet Planet. Health.

[8] Smargiassi A (2020). Exposure to ambient air pollutants and the onset of dementia in Québec, Canada. Environ. Res..

[9] Grande G, Ljungman PL, Eneroth K, Bellander T, Rizzuto D (2020). Association between cardiovascular disease and long-term exposure to air pollution with the risk of dementia. JAMA Neurol..

[10] Ilango SD (2020). The role of cardiovascular disease in the relationship between air pollution and incident dementia: a population-based cohort study. Int. J. Epidemiol..

[11] Lee M, Schwartz J, Wang Y, Dominici F, Zanobetti A (2019). Long-term effect of fine particulate matter on hospitalization with dementia. Environ. Pollut..

[12] Mortamais M (2021). Long-term exposure to ambient air pollution and risk of dementia: results of the prospective three-city study. Environ. Int..

[13] Nunez Y (2021). Fine particle exposure and clinical aggravation in neurodegenerative diseases in New York state. Environ. Health Perspect..

[14] Sullivan, K. J. et al. Ambient fine particulate matter exposure and incident mild cognitive impairment and dementia. *J. Am. Geriatr. Soc.* **69**, 2185–2194 (2021). 10.1111/jgs.17188 PMC8373708 33904156

[15] Jack CR (2010). Hypothetical model of dynamic biomarkers of the Alzheimer’s pathological cascade. Lancet Neurol..

[16] Di Q (2019). An ensemble-based model of PM2. 5 concentration across the contiguous United States with high spatiotemporal resolution. Environ. Int..

[17] Di Q (2019). Assessing NO_2_ concentration and model uncertainty with high spatiotemporal resolution across the contiguous united states using ensemble model averaging. Environ. Sci. Technol..

[18] Requia WJ (2020). An ensemble learning approach for estimating high spatiotemporal resolution of ground-level ozone in the contiguous United States. Environ. Sci. Technol..

[19] Semega, J. L., Fontenot, K. R. & Kollar, M. A. Income and poverty in the United States: 2016. *Current Population Reports* (2017).

[20] Jacob, D. J. *Introduction to atmospheric chemistry*. (Princeton University Press, 1999).

[21] Jerrett M (2009). Long-term ozone exposure and mortality. N. Engl. J. Med..

[22] Heusinkveld HJ (2016). Neurodegenerative and neurological disorders by small inhaled particles. Neurotoxicology.

[23] Peters, R., Mudway, I., Booth, A., Peters, J. & Anstey, K. J. Putting fine particulate matter and dementia in the wider context of noncommunicable disease: where are we now and what should we do next: a systematic review. *Neuroepidemiology* **55**, 253–265 (2021). 10.1159/000515394 34062541

[24] Williams M, Atkinson R, Anderson H, Kelly F (2014). Associations between daily mortality in London and combined oxidant capacity, ozone and nitrogen dioxide. Air Qual. Atmos. Health.

[25] Chen H (2017). Living near major roads and the incidence of dementia, Parkinson’s disease, and multiple sclerosis: a population-based cohort study. Lancet.

[26] Ranft U, Schikowski T, Sugiri D, Krutmann J, Krämer U (2009). Long-term exposure to traffic-related particulate matter impairs cognitive function in the elderly. Environ. Res..

[27] Tham, R. & Schikowski, T. The role of traffic-related air pollution on neurodegenerative diseases in older people: an epidemiological perspective. *J. Alzheimer’s Dis.* **79**, 949–959 (2020). 10.3233/JAD-200813 33361591

[28] Oberdörster G (2004). Translocation of inhaled ultrafine particles to the brain. Inhal. Toxicol..

[29] Chen H (2017). Exposure to ambient air pollution and the incidence of dementia: a population-based cohort study. Environ. Int..

[30] Landrigan PJ (2018). The Lancet Commission on pollution and health. Lancet.

[31] Shaffer, R. M. et al. Fine particulate matter and markers of Alzheimer’s disease neuropathology at autopsy in a community-based cohort. *J. Alzheimer’s Dis.* **79**, 1761–1773 (2021). 10.3233/JAD-201005 PMC8061707 33459717

[32] Iaccarino L (2021). Association between ambient air pollution and amyloid positron emission tomography positivity in older adults with cognitive impairment. JAMA Neurol..

[33] Younan D (2020). Particulate matter and episodic memory decline mediated by early neuroanatomic biomarkers of Alzheimer’s disease. Brain.

[34] Calderón-Garcidueñas L (2008). Long-term air pollution exposure is associated with neuroinflammation, an altered innate immune response, disruption of the blood-brain barrier, ultrafine particulate deposition, and accumulation of amyloid β-42 and α-synuclein in children and young adults. Toxicol. Pathol..

[35] Cacciottolo M (2017). Particulate air pollutants, APOE alleles and their contributions to cognitive impairment in older women and to amyloidogenesis in experimental models. Transl. Psychiatry.

[36] Levesque S, Surace MJ, McDonald J, Block ML (2011). Air pollution & the brain: subchronic diesel exhaust exposure causes neuroinflammation and elevates early markers of neurodegenerative disease. J. Neuroinflamm..

[37] Maher BA (2016). Magnetite pollution nanoparticles in the human brain. Proc. Natl. Acad. Sci..

[38] USEPA. *Air Quality - Cities and Counties*, https://www.epa.gov/air-trends/air-quality-cities-and-counties (2020).

[39] Goodman RA (2017). Prevalence of dementia subtypes in United States Medicare fee-for-service beneficiaries, 2011–2013. Alzheimer’s Dement..

[40] Kioumourtzoglou M-A (2014). Exposure measurement error in PM 2.5 health effects studies: a pooled analysis of eight personal exposure validation studies. Environ. Health.

[41] Wu X (2019). Causal inference in the context of an error prone exposure: air pollution and mortality. Ann. Appl. Stat..

[42] Zeger SL (2000). Exposure measurement error in time-series studies of air pollution: concepts and consequences. Environ. Health Perspect..

[43] Chronic Conditions Data Warehouse. *Condition Categories*, https://www2.ccwdata.org/web/guest/condition-categories (2021).

[44] Taylor DH, Fillenbaum GG, Ezell ME (2002). The accuracy of medicare claims data in identifying Alzheimer’s disease. J. Clin. Epidemiol..

[45] Taylor DH, Østbye T, Langa KM, Weir D, Plassman BL (2009). The accuracy of Medicare claims as an epidemiological tool: the case of dementia revisited. J. Alzheimer’s Dis..

[46] Shi, L. *Yearly data for NO2 and warm season ozone in the US*, 10.6084/m9.figshare.16834390 (2021).

[47] Census, U. *Summary File 3 Dataset*, https://www.census.gov/data/datasets/2000/dec/summary-file-3.html (2002).

[48] Census, U. *Summary File 1 Dataset*, https://www.census.gov/data/datasets/2010/dec/summary-file-1.html (2011).

[49] Census, U. *American Community Survey 1-Year Data (2005–2019)*, https://www.census.gov/data/developers/data-sets/acs-1year.html (2020).

[50] CDC, U. *Behavioral Risk Factor Surveillance System* https://www.cdc.gov/brfss/annual_data/annual_data.htm (2020).

[51] Area Health Resources Files, https://data.hrsa.gov/topics/health-workforce/ahrf (2019).

[52] Junninen H, Niska H, Tuppurainen K, Ruuskanen J, Kolehmainen M (2004). Methods for imputation of missing values in air quality data sets. Atmos. Environ..

[53] Hersi M (2017). Risk factors associated with the onset and progression of Alzheimer’s disease: a systematic review of the evidence. Neurotoxicology.

[54] Anstey KJ, Ee N, Eramudugolla R, Jagger C, Peters R (2019). A systematic review of meta-analyses that evaluate risk factors for dementia to evaluate the quantity, quality, and global representativeness of evidence. J. Alzheimer’s Dis..

[55] Zhang, X. Generalized estimating equations for clustered survival data. *Retrospective Theses and Dissertations,* 3063. https://lib.dr.iastate.edu/rtd/3063 (2006).

[56] Cerza, F. et al. Long-term exposure to air pollution and hospitalization for dementia in the Rome longitudinal study. *Environ. Health* **18**, 72 (2019). 10.1186/s12940-019-0511-5 PMC6689157 31399053

[57] Steenland, K. & Armstrong, B. An overview of methods for calculating the burden of disease due to specific risk factors. *Epidemiology* **17**, 512–519 (2006). 10.1097/01.ede.0000229155.05644.43 16804473

[58] Hutcheon, J. A., Chiolero, A. & Hanley, J. A. Random measurement error and regression dilution bias. *BMJ* **340**, c2289 (2010). 10.1136/bmj.c2289 20573762

[59] Fox MP, Lash TL, Greenland S (2005). A method to automate probabilistic sensitivity analyses of misclassified binary variables. Int. J. Epidemiol..

[60] Shi, L. *R code for epidemiological analyses for ADRD*, 10.6084/m9.figshare.16843528 (2021).

