# Supplementary material for: A national cohort study (2000–2018) of long-term air pollution exposure and incident dementia in older adults in the United States
Source: Nat Commun. 2021 Nov 19;12:6754. doi: 10.1038/s41467-021-27049-2 (PMC8604909; doi:10.1038/s41467-021-27049-2)
Supplement: Supplementary file 1 — Supplementary Information [file 41467_2021_27049_MOESM1_ESM.pdf]

Supplementary Information for

A national cohort study (2000-2018) of long-term air pollution exposure and incident dementia in older adults in the United States

Liuhua Shi*[#1], Kyle Steenland[#1], Haomin Li[2], Pengfei Liu[3], Yuhan Zhang[2], Robert H. Lyles[4], Weeberb J. Requia[5], Sindana D. Ilango[6], Howard H. Chang[4], Thomas Wingo[7], Rodney J. Weber[3], Joel Schwartz[8]

[#] LS and KS contributed equally.

[1] Gangarosa Department of Environmental Health, Rollins School of Public Health, Emory University, Atlanta, GA

[2] Department of Epidemiology, Rollins School of Public Health, Emory University, Atlanta, GA

[3] School of Earth and Atmospheric Sciences, Georgia Institute of Technology, Atlanta, Georgia, USA

[4] Department of Biostatistics and Bioinformatics, Rollins School of Public Health, Emory University, Atlanta, Georgia, USA

[5] School of Public Policy and Government, Fundação Getúlio Vargas, Brasília, Distrito Federal, Brazil

[6] Department of Epidemiology, School of Public Health, University of Washington, Seattle, Washington, USA

[7] Department of Neurology and Human Genetics, School of Medicine, Emory University, Atlanta, Georgia, USA

[8] Department of Environmental Health, Harvard T.H. Chan School of Public Health, Boston, Massachusetts, USA

Correspondence to: Liuhua Shi, Gangarosa Department of Environmental Health, Rollins School of Public Health, Emory University, Atlanta, Georgia, USA

E-mail: liuhua.shi@emory.edu

Supplementary tables and figures

Supplementary Table 1. Descriptive statistics for Dementia and Alzheimer's disease (AD) cases and non-cases

Supplementary Table 2. Annual $PM_{2.5}$ (g/m^3), annual NO_2 (ppb), and warm-season O_3 (ppb) levels (minimum, maximum, mean, and percentiles) over the study period for full cohort and subgroups

Supplementary Table 3. *P*-values for the null hypothesis test that the estimated associations are the same between study subgroups

Supplementary Table 4. Hazard ratios of dementia or AD per IQR increase in air pollutants among the subpopulation with a 10-year clean period, using varied lag periods

Supplementary Table 5. Hazard ratios of dementia or AD per IQR increase in air pollutants from tri-pollutant models, additionally adjusting for comorbidities

Supplementary Table 6. Hazard ratios of dementia or AD per IQR increase in air pollutants among full cohort and non-mover cohort from single pollutant and tri-pollutant models

Supplementary Table 7. Rate ratio of dementia or Alzheimer's Disease (AD) per IQR increase in air pollutants, derived from the linear rate models

Supplementary Table 8. Hazard ratio of dementia or Alzheimer's Disease (AD) per IQR increase in each pollutant, accounting for potential outcome misclassification via adjusting data for assumed sensitivity and specificity of classification (from Taylor et al. 2009).

Supplementary Table 9. Comparing the dementia and AD cohort using Medicare CCW database versus Medicare inpatient claims (2000-2016)

Supplementary Table 10. ICD-codes for dementia and Alzheimer's disease (AD) used in Medicare Chronic Conditions Warehouse (CCW) database

Supplementary Figure 1. Schematic flowchart of the study population selection.

Supplementary Table 1. Descriptive statistics for Dementia and Alzheimer's disease (AD) cases and non-cases

	Dementia cohort (N=12,233,371)				AD cohort (N= 12,456,447)			
	Dementia cases (N=2,025,130)		Non-cases (N=10,208,241)		AD cases (N=804,668)		Non-cases (N=11,651,779)	
**Variables**	**Number**	**%**	**Number**	**%**	**Number**	**%**	**Number**	**%**
**Total person-years**	15,243,922		73,791,159		5,493,543		87,784,723	
**Median follow-up years (mean)**	8.0 (7.5)		7.0 (7.2)		7.0 (6.8)		7.0 (7.5)	
**Mean age at disease**	82.5		78.7		82.4		79.4	
**Age at entry (years)**								
**65-74**	2,021,738	99.8	10,195,166	99.9	803,412	99.8	11,634,856	99.9
**75-114**	3,392	0.2	13,075	0.1	1,256	0.2	16,923	0.1
**Sex**								
**Male**	697,854	34.5	4,326,025	42.4	256,063	31.8	4,851,879	41.6
**Female**	1,327,276	65.5	5,882,216	57.6	548,605	68.2	6,799,900	58.4
**Race**								
**White**	1,825,761	90.2	9,197,441	90.1	724,301	90.0	10,489,986	90.0
**Black**	122,738	6.1	526,343	5.2	50,247	6.2	616,372	5.3
**Other[a]**	76,631	3.8	484,457	4.7	30,120	3.7	545,421	4.7
**Medicaid Eligibility**								
**Dual-Eligible**	300,659	14.8	679,876	6.7	117,021	14.5	961,597	8.3
**Non-dual Eligible**	1,724,471	85.2	9,528,365	93.3	687,647	85.5	10,690,182	91.7
**Comorbidity**								
**Diabetes**	846,906	41.8	3,586,408	35.1	310,343	38.6	4,122,971	35.4
**Hypertension**	1,866,324	92.2	8,407,182	82.3	726,959	90.3	9,546,546	81.9
**Stroke**	619,752	30.6	1,371,978	13.4	223,293	27.7	1,768,437	15.2
**Heart failure**	862,734	42.6	2,525,806	24.7	290,409	36.1	3,098,131	26.6
**No comorbidities[b]**	110,910	5.5	1,531,764	15.0	56,383	7.0	1,586,291	13.6
**Air pollutants[c]**								
**Annual $PM_{2.5}$ (μg/m^3)**	10.0 (3.5)		9.1 (3.1)		10.3 (3.5)		9.2 (3.1)	
**Annual NO_2 (ppb)**	18.2 (12.9)		16.8 (11.3)		18.7 (13.3)		17.0 (11.5)	
**Warm-season O_3 (ppb)**	43.0 (5.7)		42.6 (5.2)		43.0 (5.7)		42.6 (5.3)	

Note: [a] Other included Asian, Hispanic, American Indian or Alaskan Native, and unknown; [b] means none of the above comorbidities; [c] presented as mean concentration (interquartile range).

Supplementary Table 2. Annual $PM_{2.5}$ (µg/m^3), annual NO_2 (ppb), and warm-season O_3 (ppb) levels (minimum, maximum, mean, and percentiles) over the study period for the full cohort and subgroups

Groups	Pollutants	Min	1st	5th	25th	50th	75th	95th	99th	Max	Mean
**All**	**$PM_{2.5}$**	0.5	3.3	4.9	7.7	9.2	10.8	13.5	15.1	25.9	9.3
	**NO_2**	0.5	5.2	8.0	10.5	14.8	22.0	34.0	41.8	122.2	17.1
	**O_3**	19.8	29.1	33.4	40.0	42.7	45.4	51.2	57.3	77.7	42.6
**Male**	**$PM_{2.5}$**	0.5	3.2	4.7	7.5	9.1	10.7	13.4	15.0	25.9	9.1
	**NO_2**	0.8	5.1	6.9	10.3	14.6	21.6	33.6	41.5	120.7	16.8
	**O_3**	19.8	29.1	33.3	40.0	42.7	45.4	51.5	57.7	77.6	42.7
**Female**	**$PM_{2.5}$**	0.6	3.4	5.0	7.7	9.3	10.9	13.6	15.1	24.4	9.3
	**NO_2**	0.5	5.2	7.0	10.6	15.0	22.3	34.3	42.0	122.2	17.2
	**O_3**	29.1	33.5	40.1	42.7	45.3	51.0	57.0	20.4	77.7	42.6
**White**	**$PM_{2.5}$**	0.5	3.3	4.8	7.6	9.2	10.8	13.4	15.0	25.9	9.2
	**NO_2**	0.5	5.1	6.9	10.4	14.5	21.4	33.4	41.5	122.2	16.7
	**O_3**	20.4	29.2	33.5	40.0	42.7	45.3	51.2	56.9	77.7	42.6
**Black**	**$PM_{2.5}$**	1.7	5.8	7.2	8.8	10.0	11.5	13.9	15.3	24.4	10.2
	**NO_2**	0.9	6.0	7.5	11.6	19.3	27.4	36.9	42.7	74.6	20.2
	**O_3**	21.1	29.5	35.6	40.4	42.7	45	48.3	53.9	77.6	42.6
**Other**	**$PM_{2.5}$**	1.0	3.2	4.5	7.7	9.3	10.9	14.1	17.1	24.5	9.3
	**NO_2**	1.6	5.3	7.8	13.1	20	27.5	39.0	46.5	114.5	21.1
	**O_3**	19.8	27.3	29.7	39.0	42.9	46.5	54.2	61.6	75.3	42.7
**Non-Medicaid**	**$PM_{2.5}$**	0.5	3.3	4.8	7.6	9.2	10.8	13.5	15.0	24.5	9.2
	**NO_2**	0.5	5.2	7.0	10.5	14.9	21.9	33.8	41.5	122.2	17.0
	**O_3**	20.4	29.2	33.6	40.1	42.7	45.4	51.2	56.8	77.7	42.7
**Medicaid**	**$PM_{2.5}$**	1.5	3.4	5.1	7.9	9.5	11.2	13.9	16.3	25.9	9.6
	**NO_2**	0.9	4.9	6.6	9.9	14.7	23.8	37.1	44.4	81.0	17.6
	**O_3**	19.8	27.8	31.8	39.7	42.5	45.3	51.7	60.6	77.4	42.4
**Age < 75**	**$PM_{2.5}$**	0.5	3.3	4.9	7.7	9.2	10.8	13.5	15.1	25.9	9.2
	**NO_2**	0.5	5.2	6.9	10.5	14.8	22.0	34.0	41.8	122.2	17.1
	**O_3**	19.8	29.1	33.4	40.0	42.7	45.4	51.2	57.3	77.7	42.6
**Age ≥ 75**	**$PM_{2.5}$**	1.9	3.9	5.9	8.3	9.8	11.5	14.4	17.3	23.5	9.9
	**NO_2**	1.9	5.8	7.8	13.2	19.9	27.3	38.7	46.0	74.6	21.0
	**O_3**	20.5	27.7	30.2	39.5	42.7	45.3	51.0	57.9	70.5	42.1
**Density Q1**	**$PM_{2.5}$**	0.5	2.7	3.6	6.6	9.7	10.2	12.6	14	21.9	8.4
	**NO_2**	0.8	4.0	5.7	7.7	9.6	12.1	17.2	22.6	67.3	10.3
	**O_3**	24.4	32.6	36.0	40.5	42.9	45.5	50.8	54.3	73.6	43.1
**Density Q2**	**$PM_{2.5}$**	0.8	3.6	4.8	7.5	9.2	10.8	13.4	14.8	24.4	9.2
	**NO_2**	1.1	5.4	7.2	9.9	12.4	15.8	22.1	28.5	75.8	12.4
	**O_3**	20.4	30.3	34.7	40.4	42.9	45.3	50.9	58.9	76.0	42.9
**Density Q3**	**$PM_{2.5}$**	1.7	4.6	6.0	7.9	9.3	11.0	13.6	15.0	24.4	9.5
	**NO_2**	1.6	7.2	9.7	13.7	17.9	22.8	30.8	37.4	68.7	18.7
	**O_3**	23.6	29.8	33.1	39.8	42.7	45.3	51.2	58.5	77.7	42.5
**Density Q4**	**$PM_{2.5}$**	0.6	5.5	6.6	8.3	9.7	11.4	14.2	16.2	25.9	9.7
	**NO_2**	0.5	9.2	13.1	19.6	25.4	31.8	40.5	47.8	122.2	25.4
	**O_3**	19.8	27.9	30.2	39.3	42.3	45.2	52.2	58.5	72.9	42.3

Supplementary Table 3. *P*-values for the null hypothesis test that the estimated associations are the same between study subgroups

	Dementia			AD		
**Subgroups**	**$PM_{2.5}$**	**NO_2**	**O_3**	**$PM_{2.5}$**	**NO_2**	**O_3**
Male	Reference	Reference	Reference	Reference	Reference	Reference
Female	0.64	0.003	0.05	0.68	0.33	0.81
White	Reference	Reference	Reference	Reference	Reference	Reference
Black	<0.001	<0.001	0.04	0.50	<0.001	0.46
Other	0.57	0.12	0.38	0.73	0.40	<0.001
Non-Medicaid	Reference	Reference	Reference	Reference	Reference	Reference
Medicaid	<0.001	<0.001	<0.001	<0.001	0.006	0.03
Age<75	Reference	Reference	Reference	Reference	Reference	Reference
Age≥75	<0.001	<0.001	0.10	<0.001	<0.001	0.03
Density Q1	Reference	Reference	Reference	Reference	Reference	Reference
Density Q2	0.001	<0.001	0.06	0.006	<0.001	0.11
Density Q3	<0.001	<0.001	0.003	<0.001	<0.001	0.01
Density Q4	<0.001	0.50	0.01	<0.001	<0.001	0.01

Supplementary Table 4. Hazard ratios of dementia or AD per IQR increase in air pollutants among the subpopulation with a 10-year clean period, using varied lag periods

	$PM_{2.5}$	NO_2	O_3
**Dementia – Single pollutant**			
Lag 0	1.062 (1.055, 1.069)	1.029 (1.022, 1.036)	1.003 (0.999, 1.006)
Lag 1	1.052 (1.046, 1.059)	1.021 (1.015, 1.028)	0.998 (0.994, 1.001)
Lag 5	1.042 (1.037, 1.046)	1.018 (1.012, 1.023)	1.004 (1.001, 1.007)
Lag 10	1.028 (1.025, 1.032)	1.011 (1.007, 1.015)	1.003 (1.000, 1.005)
Average (lags 0-5)	1.053 (1.047, 1.059)	1.022 (1.015, 1.029)	0.999 (0.995, 1.002)
**AD – Single pollutant**			
Lag 0	1.096 (1.082, 1.111)	1.076 (1.062, 1.089)	0.991 (0.984, 0.999)
Lag 1	1.091 (1.079, 1.103)	1.064 (1.052, 1.076)	0.986 (0.979, 0.992)
Lag 5	1.077 (1.068, 1.087)	1.052 (1.042, 1.063)	0.996 (0.991, 1.002)
Lag 10	1.066 (1.058, 1.073)	1.042 (1.033, 1.050)	0.992 (0.987, 0.998)
Average (lags 0-5)	1.079 (1.070, 1.088)	1.049 (1.039, 1.059)	0.996 (0.991, 1.001)
**Dementia – Multi-pollutant**			
Lag 0	1.060 (1.053, 1.067)	1.016 (1.009, 1.023)	0.995 (0.991, 0.998)
Lag 1	1.053 (1.047, 1.060)	1.012 (1.005, 1.018)	0.990 (0.987, 0.994)
Lag 5	1.041 (1.036, 1.047)	1.007 (1.001, 1.012)	0.997 (0.994, 0.999)
Lag 10	1.030 (1.026, 1.035)	1.001 (0.996, 1.006)	0.995 (0.993, 0.998)
Average (lags 0-5)	1.055 (1.048, 1.062)	1.009 (1.002, 1.016)	0.991 (0.987, 0.994)
**AD – Multi-pollutant**			
Lag 0	1.090 (1.075, 1.104)	1.063 (1.050, 1.077)	0.976 (0.968, 0.983)
Lag 1	1.090 (1.077, 1.103)	1.053 (1.041, 1.066)	0.970 (0.964, 0.977)
Lag 5	1.078 (1.067, 1.088)	1.038 (1.027, 1.048)	0.979 (0.974, 0.985)
Lag 10	1.073 (1.065, 1.081)	1.022 (1.013, 1.030)	0.973 (0.968, 0.978)
Average (lags 0-5)	1.078 (1.068, 1.088)	1.033 (1.023, 1.043)	0.981 (0.976, 0.986)

Note: All hazard ratios were calculated using the same IQRs with the main analysis.

Supplementary Table 5. Hazard ratios of dementia or AD per IQR increase in air pollutants from tri-pollutant models, additionally adjusting for comorbidities

Outcome	Models	$PM_{2.5}$	NO_2	O_3
**Dementia**	Main model + diabetes	1.058 (1.052, 1.064)	1.004 (0.997, 1.010)	0.993 (0.990, 0.996)
	Main model + hypertension	1.051 (1.045, 1.057)	1.014 (1.008, 1.021)	0.992 (0.988, 0.995)
	Main model + stroke	1.051 (1.046, 1.057)	1.013 (1.007, 1.020)	0.991 (0.988, 0.994)
	Main model + heart failure	1.052 (1.046, 1.058)	1.003 (0.997, 1.009)	0.992 (0.988, 0.995)
	Main model among no comorbidities	1.061 (1.048, 1.075)	0.922 (0.910, 0.935)	1.029 (1.021, 1.037)
**AD**	Main model + diabetes	1.077 (1.069, 1.084)	1.022 (1.014, 1.030)	0.983 (0.979, 0.988)
	Main model + hypertension	1.072 (1.064, 1.080)	1.027 (1.019, 1.036)	0.983 (0.978, 0.987)
	Main model + stroke	1.071 (1.064, 1.079)	1.027 (1.018, 1.035)	0.983 (0.978, 0.987)
	Main model + heart failure	1.074 (1.066, 1.081)	1.022 (1.014, 1.031)	0.983 (0.978, 0.987)
	Main model among no comorbidities	1.062 (1.044, 1.080)	0.900 (0.882, 0.917)	1.021 (1.011, 1.032)

Supplementary Table 6. Hazard ratios of dementia or AD per IQR increase in air pollutants among full cohort and non-mover cohort from single pollutant and tri-pollutant models

Outcome	Model	$PM_{2.5}$	NO_2	O_3
Dementia		**Full cohort (N=12,233,371)**		
	**Single pollutant**	1.061 (1.056, 1.067)	1.035 (1.028, 1.042)	1.002 (0.998, 1.005)
	**Tri-pollutant**	1.060 (1.054, 1.066)	1.019 (1.012, 1.026)	0.990 (0.987, 0.993)
		**Non-mover (N=10,410,080)**		
	**Single pollutant**	1.049 (1.043, 1.055)	1.048 (1.041, 1.055)	0.986 (0.983, 0.990)
	**Tri-pollutant**	1.057 (1.051, 1.062)	1.059 (1.052, 1.066)	1.000 (0.996, 1.003)
AD		**Full cohort (N=12,456,447)**		
	**Single pollutant**	1.078 (1.071, 1.086)	1.050 (1.042, 1.059)	0.999 (0.994,1.003)
	**Tri-pollutant**	1.078 (1.070, 1.086)	1.031 (1.023, 1.039)	0.982 (0.977, 0.986)
		**Non-mover (N=10,487,122)**		
	**Single pollutant**	1.064 (1.056, 1.072)	1.065 (1.056, 1.074)	0.975 (0.970, 0.980)
	**Tri-pollutant**	1.072 (1.064, 1.080)	1.078 (1.069, 1.087)	0.994 (0.989, 0.999)

Supplementary Methods.

We conducted sensitivity analyses to estimate the effect of possible outcome misclassification in two ways:

First, we fit linear regression models for the rate of dementia or AD (events/person-time) with a generalized estimating equation, which resulted in an estimate of the additive effect less sensitive to bias. The advantage is that in a linear model random misclassification of the outcome would tend to absorb into the residual errors of the linear model for the true counts so that the outcome misclassification should be less likely to produce markedly biased coefficient estimates (e.g., Hutcheon et al., 2020)[1]. In the linear rate model, the outcome is rate, and the coefficient of exposure is the incremental probability of the event (i.e., the increase in rate) for a unit change in exposure.

Second, we considered the possible effect of outcome misclassification, in a manner similar to the methods described by Fox et al. (2005)[2]. We used estimates of misclassification from Taylor et al. (2009)[3] and adjusted the observed case counts for each zip code in the stratified Poisson model to match up with the expected true values given pre-specified values for sensitivity and specificity for the outcome classification.

We first reduced the number of observations in the stratified Poisson model by restricting to stratification by age and race, rather than all the co-variates included in the original Cox model, in order to avoid smaller strata with 0 disease counts. We then switched focus from person-time to case counts, in order to adjust the observed proportion of cases via our correction for misclassification. We also restricted the data so that each person was associated with only one ZIP code, which was the ZIP code where he/she had lived the longest during the follow-up period. Then each person was assigned the exposure level of that ZIP code. We used the log of the number of people in each ZIP code as the offset associated with the corresponding record in the regression analysis. Using this new data, the original Cox model exposure coefficients, as well as the new exposure coefficient using a simplified Poisson model based on people rather than person-time, are shown in Supplementary Table 8, based on the tri-pollutants models for both dementia and AD. Finally, we corrected the data for misclassification using the observed error-prone case counts of dementia and AD in each ZIP code, and the sensitivity and specificity for disease classification taken from Taylor et al. (2009), who compared Medicare diagnoses to a gold standard based on clinical diagnoses for 794 members of the Aging Demographics and Memory Study (ADAMS). Taylor et al. (2009)[3] estimated the sensitivity and specificity of Medicare claims as 0.85 and 0.89 for dementia, and 0.64 and 0.95 for AD. The correction was done using the formula below (Supplementary Equation 1) (Bross 1954)[4]:

$$\hat{p}_i = (\hat{p}_i^* + SP - 1)/(SE + SP - 1) \qquad (1)$$

where $\hat{p}_i^*$ is the observed proportion of cases in each ZIP code i, SP and SE are specificity and sensitivity taken from Taylor et al. (2009)[3], and $\hat{p}_i$ is the corrected proportion of cases in each ZIP code i. As the formula above yields a negative number of corrected cases when the proportion of observed cases is less than 1-SP, we restricted this correction for dementia or AD to ZIP codes where the observed proportion was greater than 1-SP (11% for dementia, 5% for AD); for strata with an observed proportion less than 1-SP, the corrected case count was set to 0. Under these conditions, Supplementary Table 8 gives the corrected values of the exposure coefficients for both dementia and AD, Corrected HR values from the original Cox model were

obtained by taking the ratio of the corrected vs uncorrected exposure coefficients from the Poisson model described above (from models 3 and 2 in Supplementary Table 8), and then multiplying the exposure coefficient from the original Cox model by that ratio, and obtaining the HR for an IQR increase in $PM_{2.5}$ using that corrected Cox model coefficient.

Supplementary Table 7. Rate ratio of dementia or Alzheimer's Disease (AD) per IQR increase in air pollutants, derived from the linear rate models.

	**Cox model**	**Linear rate model**
**Dementia**		
$PM_{2.5}$	**1.060** (1.054, 1.066)	**1.067** (1.062, 1.071)
NO_2	**1.019** (1.012, 1.026)	**1.064** (1.058, 1.069)
O_3	**0.990** (0.987, 0.993)	**0.990** (0.987, 0.993)
**AD**		
$PM_{2.5}$	**1.078** (1.070, 1.086)	**1.105** (1.098, 1.113)
NO_2	**1.031** (1.023, 1.039)	**1.071** (1.063, 1.080)
O_3	**0.982** (0.977, 0.986)	**0.978** (0.974, 0.983)

Note: The baseline incidence rate of dementia or AD was calculated based on the number of events and total person years in Table 1.

Supplementary Table 8. Hazard ratio of dementia or Alzheimer's Disease (AD) per IQR increase in each pollutant, accounting for potential outcome misclassification via adjusting data for assumed sensitivity and specificity of classification (from Taylor et al. 2009[3]).

	**Original Cox model (model 1)**	**Poisson model with number of people instead of person-time* (model 2)***	**Corrected hazard ratio from Poisson model with number of people instead of person-time (model 3)****	**Corrected hazard ratio from original Cox model (model 4)*****
**Dementia**				
$PM_{2.5}$	**1.060** (1.054, 1.066)	1.063 (1.060, 1.066)	1.084 (1.077, 1.090)	**1.081** (1.074, 1.086)
NO_2	**1.019** (1.012, 1.026)	1.030 (1.027, 1.033)	1.041 (1.033, 1.048)	**1.026** (1.021, 1.030)
O_3	**0.990** (0.987, 0.993)	0.979 (0.977, 0.980)	0.968 (0.965, 0.972)	**0.985** (0.983, 0.987)
**AD**				
$PM_{2.5}$	**1.078** (1.070, 1.086)	1.102 (1.097, 1.107)	1.152 (1.139, 1.165)	**1.115** (1.106, 1.125)
NO_2	**1.031** (1.023, 1.039)	1.055 (1.050, 1.060)	1.091 (1.082, 1.101)	**1.051** (1.046, 1.056)
O_3	**0.982** (0.977, 0.986)	0.963 (0.960, 0.966)	0.946 (0.940, 0.951)	**0.973** (0.970, 0.976)

*model with stratification by age and race, and exposure assigned to ZIP code of longest residence during follow-up period. Confidence intervals are overly narrow for corrected hazard as they do not reflect uncertainty due to the degree of correction.
** same model as model 2 but corrected for misclassification described in text above.
*** Original coefficient from Cox model, corrected by multiplying the ratio of the exposure coefficients for model 3/model 2 by the original Cox model exposure coefficient, then multiplying that corrected coefficient by the IQR and exponentiating to get the corrected HR. 95% CIs for models 3 and 4 were calculated from 50 bootstrap samples having the same data size with the modeled dataset.

Supplementary Table 9. Comparing the dementia and AD cohort using Medicare CCW database versus Medicare inpatient claims (2000-2016)

	AD	Dementia
Current study using Medicare CCW database (2000-2016)		
Number of claims	5,646,187	11,121,272
Total person-years	403,149,214	379,921,997
Previous study using Medicare inpatient claims[5] (2000-2016)		
Number of admissions	2,490,431	1,233,132
Total person-years	475,820,277	478,636,053

Supplementary Table 10. ICD-codes for dementia and Alzheimer's disease (AD) used in Medicare Chronic Conditions Warehouse (CCW) database

	**Dementia**	**AD**
ICD-9	DX 331.0, 331.11, 331.19, 331.2, 331.7, 290.0, 290.10, 290.11, 290.12, 290.13, 290.20, 290.21, 290.3, 290.40, 290.41, 290.42, 290.43, 294.0, 294.10, 294.11, 294.20, 294.21, 294.8, 797 (any DX on the claim)	DX 331.0 (any DX on the claim)
ICD-10	DX F01.50, F01.51, F02.80, F02.81, F03.90, F03.91, F04, G13.2, G13.8, F05, F06.1, F06.8, G30.0, G30.1, G30.8, G30.9, G31.1, G31.2, G31.01, G31.09, G91.4, G94, R41.81, R54 (any DX on the claim)	DX G30.0, G30.1, G30.8, G30.9 (any DX on the claim)

Note: DX denotes diagnosis; ICD-10 codes are effective 10/2015.

Supplementary Figure 1. Schematic flowchart of the study population selection.

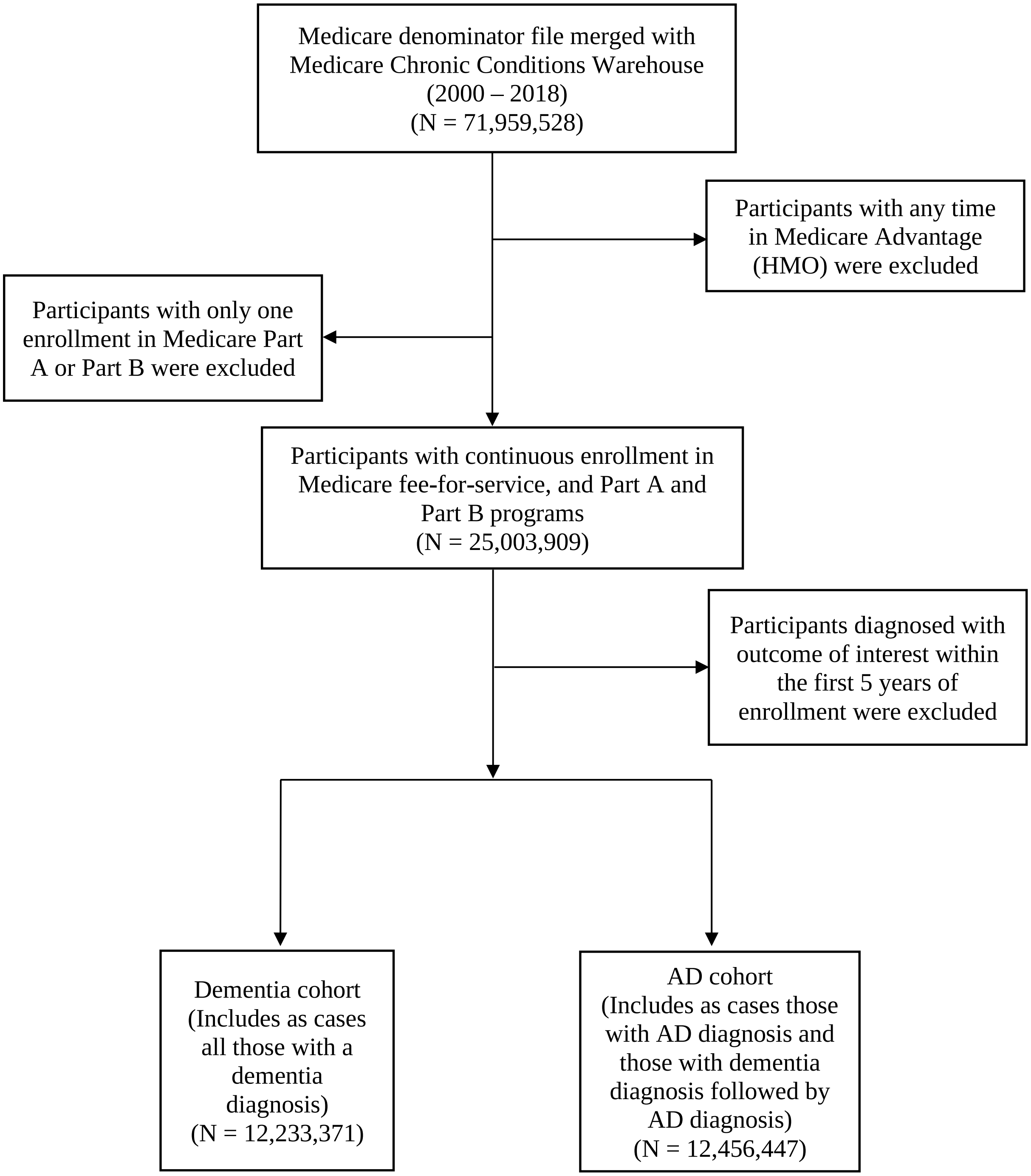

Supplementary References

1 Hutcheon, J. A., Chiolero, A. & Hanley, J. A. Random measurement error and regression dilution bias. *Bmj* 340 (2010).

2 Fox, M. P., Lash, T. L. & Greenland, S. A method to automate probabilistic sensitivity analyses of misclassified binary variables. *International journal of epidemiology* 34, 1370-1376 (2005).

3 Taylor Jr, D. H., Østbye, T., Langa, K. M., Weir, D. & Plassman, B. L. The accuracy of Medicare claims as an epidemiological tool: the case of dementia revisited. *Journal of Alzheimer's Disease* 17, 807-815 (2009).

4 Bross, I. Misclassification in 2 x 2 tables. *Biometrics* 10, 478-486 (1954).

5 Shi, L. *et al.* Long-term effects of PM2· 5 on neurological disorders in the American Medicare population: a longitudinal cohort study. *The Lancet Planetary Health* 4, e557-e565 (2020).
