# Supplementary material for: A national cohort study (2000–2018) of long-term air pollution exposure and incident dementia in older adults in the United States
Source: Nat Commun. 2021 Nov 19;12:6754. doi: 10.1038/s41467-021-27049-2 (PMC8604909; doi:10.1038/s41467-021-27049-2)
Supplement: Supplementary file 2 — Reporting summary [file 41467_2021_27049_MOESM2_ESM.pdf]

nature research

Corresponding author(s): Liuhua Shi

Last updated by author(s): October 18, 2021

# Reporting Summary

Nature Research wishes to improve the reproducibility of the work that we publish. This form provides structure for consistency and transparency in reporting. For further information on Nature Research policies, see our Editorial Policies and the Editorial Policy Checklist.

## Statistics

For all statistical analyses, confirm that the following items are present in the figure legend, table legend, main text, or Methods section.

| n/a | Confirmed | |
|---|---|---|
| ☐ | ☒ | The exact sample size (*n*) for each experimental group/condition, given as a discrete number and unit of measurement |
| ☐ | ☒ | A statement on whether measurements were taken from distinct samples or whether the same sample was measured repeatedly |
| ☐ | ☒ | The statistical test(s) used AND whether they are one- or two-sided<br>*Only common tests should be described solely by name; describe more complex techniques in the Methods section.* |
| ☐ | ☒ | A description of all covariates tested |
| ☒ | ☐ | A description of any assumptions or corrections, such as tests of normality and adjustment for multiple comparisons |
| ☐ | ☒ | A full description of the statistical parameters including central tendency (e.g. means) or other basic estimates (e.g. regression coefficient) AND variation (e.g. standard deviation) or associated estimates of uncertainty (e.g. confidence intervals) |
| ☐ | ☒ | For null hypothesis testing, the test statistic (e.g. *F*, *t*, *r*) with confidence intervals, effect sizes, degrees of freedom and *P* value noted<br>*Give P values as exact values whenever suitable.* |
| ☒ | ☐ | For Bayesian analysis, information on the choice of priors and Markov chain Monte Carlo settings |
| ☒ | ☐ | For hierarchical and complex designs, identification of the appropriate level for tests and full reporting of outcomes |
| ☐ | ☒ | Estimates of effect sizes (e.g. Cohen's *d*, Pearson's *r*), indicating how they were calculated |

*Our web collection on statistics for biologists contains articles on many of the points above.*

## Software and code

Policy information about availability of computer code

| | |
|---|---|
| Data collection | The health data relied on already existent Medicare administrative data collected by the Centers for Medicare & Medicaid Services (CMS). The other data were directly downloaded from open sources. No software or code was involved in data collection. |
| Data analysis | All computational analyses were run on the Rollins High-Performance Computing (HPC) Cluster at Emory University. R software, version 4.0.2, was used for all analyses. The R codes for the epidemiological analyses are publicly available from https://doi.org/10.6084/m9.figshare.16843528. |

For manuscripts utilizing custom algorithms or software that are central to the research but not yet described in published literature, software must be made available to editors and reviewers. We strongly encourage code deposition in a community repository (e.g. GitHub). See the Nature Research guidelines for submitting code & software for further information.

## Data

Policy information about availability of data

All manuscripts must include a data availability statement. This statement should provide the following information, where applicable:

- Accession codes, unique identifiers, or web links for publicly available datasets
- A list of figures that have associated raw data
- A description of any restrictions on data availability

Ensemble-based PM2.5 data that support the findings of this study are available from https://doi.org/10.7927/0rvr-4538, NO2 and O3 data are available from https://doi.org/10.6084/m9.figshare.16834390. Behavioral risk factors are available from https://www.cdc.gov/brfss/annual_data/annual_data.htm; socioeconomic status data are available from https://www.census.gov/data/datasets/2000/dec/summary-file-3.html, https://www.census.gov/data/datasets/2010/dec/summary-file-1.html, and https://www.census.gov/data/developers/data-sets/acs-1year.html; health care capacity data are available from https://data.hrsa.gov/topics/health-workforce/ahrf. The rules governing the Medicare dataset prohibit any sharing of the health datasets being used for our epidemiologic research. Restricted

by our Data Use Agreement with the U.S. Centers for Medicare & Medicaid Services, the Medicare data that support the findings of this study are neither sharable nor publicly available. Academic and non-profit researchers who are interested in using Medicare data should contact the U.S. Centers for Medicare & Medicaid Services directly to obtain their own datasets upon completion of a Data Use Agreement. The source data underlying all figures are provided in the Source Data file.

# Field-specific reporting

Please select the one below that is the best fit for your research. If you are not sure, read the appropriate sections before making your selection.

☐ Life sciences ☐ Behavioural & social sciences ☒ Ecological, evolutionary & environmental sciences

For a reference copy of the document with all sections, see nature.com/documents/nr-reporting-summary-flat.pdf

# Ecological, evolutionary & environmental sciences study design

All studies must disclose on these points even when the disclosure is negative.

| | |
|---|---|
| Study description | We constructed two national population-based cohorts of those aged 65 and above from the Medicare Chronic Conditions Warehouse (2000-2018), including doctor visits, to investigate the impact of long-term exposure to ambient fine particulate matter (PM2.5), nitrogen dioxide (NO2), and ozone (O3) on dementia and AD incidence, respectively. Data were analyzed via Cox proportional hazards regression. |
| Research sample | This study population was based on the 100% Medicare claims data in Medicare Chronic Conditions Warehouse (CCW). All eligible samples were finally involved in our study. This study finally included 12,233,371 individuals in the dementia cohort and 12,456,447 individuals in the Alzheimer's disease cohort. |
| Sampling strategy | All Medicare beneficiaries aged 65 and over who were always enrolled (1) in Medicare Fee-for-Service program; and (2) in both Medicare Part A (hospital insurance) and Part B (medical insurance) in the contiguous United States between 2000 and 2018 were selected in our study cohort. No further sampling procedure was conducted. This study finally included 12,233,371 individuals in the dementia cohort and 12,456,447 individuals in the Alzheimer's disease cohort, which allow very large-scale population-based cohort study. |
| Data collection | The health data relied on already existent Medicare administrative data collected by the Centers for Medicare & Medicaid Services (CMS) and derived from reimbursement information or the payment of bills. The data include all U.S. residents that are eligible to enter the Medicare program after they turn 65 years of age. The pollution data were derived using spatiotemporal ensemble models that integrated three different machine learning algorithms, which could be publicly available from https://doi.org/10.7927/0rvr-4538. |
| Timing and spatial scale | The Medicare administrative data collected beneficiaries' information annually, e.g., age, Medicaid eligibility, and ZIP code of residence for each Medicare beneficiary were updated annually. We acquired the nationwide Medicare data across the contiguous US from 2000-2018. The smallest geographic unit of these Medicare data was ZIP code, i.e., we know in which ZIP code each Medicare enrollee resides for each calendar year. |
| Data exclusions | We excluded those with any time in Medicare Advantage (HMO) over the study period since claim records are not available for these beneficiaries and excluded those only enrolled in Medicare Part A or Part B. Without these restrictions we can miss diagnoses of interest for the study population, i.e. we aim to make sure that we have access to all their claims for each subject included in our cohort so that we can better capture their first diagnosis and investigate disease incidence. |
| Reproducibility | All attempts to repeat the experiment were successful. |
| Randomization | NA. This study is an observational study, and we didn't perform further sampling or randomizing |
| Blinding | This is an observational study, and blinding is not relevant. |

Did the study involve field work? ☐ Yes ☒ No

# Reporting for specific materials, systems and methods

We require information from authors about some types of materials, experimental systems and methods used in many studies. Here, indicate whether each material, system or method listed is relevant to your study. If you are not sure if a list item applies to your research, read the appropriate section before selecting a response.

## Materials & experimental systems

| n/a | Involved in the study | |
|---|---|---|
| ☒ | ☐ | Antibodies |
| ☒ | ☐ | Eukaryotic cell lines |
| ☒ | ☐ | Palaeontology and archaeology |
| ☒ | ☐ | Animals and other organisms |
| ☐ | ☒ | Human research participants |
| ☒ | ☐ | Clinical data |
| ☒ | ☐ | Dual use research of concern |

## Methods

| n/a | Involved in the study | |
|---|---|---|
| ☒ | ☐ | ChIP-seq |
| ☒ | ☐ | Flow cytometry |
| ☒ | ☐ | MRI-based neuroimaging |

# Human research participants

Policy information about studies involving human research participants

| | |
|---|---|
| Population characteristics | There were 12.2 and 12.4 million people in the dementia and AD cohorts, respectively. In both cohorts, about 59% were female, and most of the studied subjects (78.5% and 78.1% for dementia and AD, respectively) entered the cohorts between ages 65 and 74. The median follow-up was 7 years in both cohorts. A majority of the study population had a co-morbidity at some point during follow-up. 16.6% developed dementia (~2.0 million cases), 6.5% developed AD (~0.8 million cases). |
| Recruitment | This is a secondary data analysis from existing medical records collected by the Centers for Medicare & Medicaid Services (CMS). |
| Ethics oversight | This study was approved by the Institutional Review Board of Emory University and a waiver of informed consent was granted |

Note that full information on the approval of the study protocol must also be provided in the manuscript.
